# Peak wall rupture index is associated with risk of rupture of abdominal aortic aneurysms, independent of size and sex

**DOI:** 10.1093/bjs/znae125

**Published:** 2024-05-24

**Authors:** Antti Siika, Mareia Talvitie, Moritz Lindquist Liljeqvist, Marko Bogdanovic, T Christian Gasser, Rebecka Hultgren, Joy Roy

**Affiliations:** Department of Molecular Medicine and Surgery, Karolinska Institute, Stockholm, Sweden; Department of Molecular Medicine and Surgery, Karolinska Institute, Stockholm, Sweden; Department of Vascular Surgery, Karolinska University Hospital, Stockholm, Sweden; Department of Molecular Medicine and Surgery, Karolinska Institute, Stockholm, Sweden; Department of Vascular Surgery, Karolinska University Hospital, Stockholm, Sweden; Department of Molecular Medicine and Surgery, Karolinska Institute, Stockholm, Sweden; Department of Vascular Surgery, Karolinska University Hospital, Stockholm, Sweden; KTH Solid Mechanics, Department of Engineering Mechanics, School of Engineering Sciences, KTH Royal Institute of Technology, Stockholm, Sweden; Faculty of Health Sciences, University of Southern Denmark, Odense, Denmark; Department of Molecular Medicine and Surgery, Karolinska Institute, Stockholm, Sweden; Department of Vascular Surgery, Karolinska University Hospital, Stockholm, Sweden; Department of Molecular Medicine and Surgery, Karolinska Institute, Stockholm, Sweden; Department of Vascular Surgery, Karolinska University Hospital, Stockholm, Sweden

## Abstract

**Background:**

Information on the predictive determinants of abdominal aortic aneurysm rupture from CT angiography are scarce. The aim of this study was to investigate biomechanical parameters in abdominal aortic aneurysms and their association with risk of subsequent rupture.

**Methods:**

In this retrospective study, the digital radiological archive was searched for 363 patients with ruptured abdominal aortic aneurysms. All patients who underwent at least one CT angiography examination before aneurysm rupture were included. CT angiography results were analysed to determine maximum aneurysm diameter, aneurysm volume, and biomechanical parameters (peak wall stress and peak wall rupture index). In the primary survival analysis, patients with abdominal aortic aneurysms less than 70 mm were considered. Sensitivity analyses including control patients and abdominal aortic aneurysms of all sizes were performed.

**Results:**

A total of 67 patients who underwent 109 CT angiography examinations before aneurysm rupture were identified. The majority were men (47, 70%) and the median age at the time of CTA examination was 77 (71–83) years. The median maximum aneurysm diameter was 56 (interquartile range 46–65) mm and the median time to rupture was 2.13 (interquartile range 0.64–4.72) years. In univariable analysis, maximum aneurysm diameter, aneurysm volume, peak wall stress, and peak wall rupture index were all associated with risk of rupture. Women had an increased HR for rupture when adjusted for maximum aneurysm diameter or aneurysm volume (HR 2.16, 95% c.i. 1.23 to 3.78 (*P* = 0.007) and HR 1.92, 95% c.i. 1.06 to 3.50 (*P* = 0.033) respectively). In multivariable analysis, the peak wall rupture index was associated with risk of rupture. The HR for peak wall rupture index was 1.05 (95% c.i. 1.03 to 1.08) per % (*P* < 0.001) when adjusted for maximum aneurysm diameter and 1.05 (95% c.i. 1.02 to 1.08) per % (*P* < 0.001) when adjusted for aneurysm volume.

**Conclusion:**

Biomechanical factors appear to be important in the prediction of abdominal aortic aneurysm rupture. Women are at increased risk of rupture when adjustments are made for maximum aneurysm diameter alone.

## Teaser

This study investigated factors associated with risk of rupture of abdominal aortic aneurysms from CT angiography imaging before rupture. Biomechanical parameters, particularly peak wall rupture index, are associated with an increased risk of rupture, independent of size and sex, and women suffer an increased risk of rupture.

## Introduction

Surgical treatment for abdominal aortic aneurysms (AAAs) is considered when the estimated risk of rupture is larger than the risks associated with the operation. Treatment guidelines for AAAs state that the maximum aneurysm diameter (Dmax) should be used as a marker for the risk of rupture and intervention is recommended at 55 mm or possibly 50 mm in women^1,2^. These guidelines are based on several randomized trials^3–6^. In men with AAAs detected during screening, the policy is efficient in preventing ruptures, with rates below 0.5% per year^7^. Ruptures of small AAAs are still seen. The UK Small Aneurysm Trial reported yearly rupture rates between 1.6% and 3.2%^8^. In the MA3RS trial, 5% of participants had AAAs that ruptured during surveillance^9^. In patients who experience ruptures, a third of ruptures occur in patients with known AAAs^10^. Certain patient populations, such as women and smokers, seem to be at a particular risk for rupture of small AAAs^11,12^. However, the majority of aneurysms rupture at diameters considerably larger than 55 mm^13–15^. Altogether, this indicates that when rupture prediction solely relies on measurement of the Dmax, some ruptures will occur before scheduled elective repair and some patients will have ruptures that are repaired long before rupture would have occurred.

The biomechanical hypothesis states that an aneurysm ruptures when wall stress exceeds wall strength and several biomechanical markers have been retrospectively validated. Peak wall stress (PWS) represents the highest wall stress in an AAA and peak wall rupture index (PWRI) represents the highest ratio of wall stress to wall strength in an AAA. Both are increased in ruptured aneurysms compared with stable controls^16,17^. In a recent meta-analysis, PWRI, but not PWS, was elevated in ruptured AAAs when compared with diameter-matched controls^18^. These studies have served as a proof-of-concept, but do not represent the clinical situation, where the goal is to estimate future rupture risk of AAAs that have not yet ruptured. PWRI has also been suggested to be elevated in aneurysms before rupture^19,20^, but this has only been evaluated in smaller cohorts of patients with pre-rupture AAAs (<20 patients), where patients have been grouped based on time to rupture, rather than utilizing survival analysis methods.

The aim of this study was to characterize biomechanical determinants in radiological examinations performed for patients who will later experience AAA rupture.

## Methods

### Patient cohort

#### Patients with imaging before aneurysm rupture

Patients were retrospectively screened for inclusion either from the Stockholm Aneurysm Rupture Cohort, which consists of patients who presented with ruptured AAAs to any hospital in Stockholm County between 2009 and 2013 (details about this cohort were published previously^14^), or from patients who presented with ruptured AAAs to Karolinska University Hospital between 2014 and 2018 (*Fig. S1*). The hospital digital archive for radiological images was retrospectively searched and all patients who underwent at least one CT angiography (CTA) examination before aneurysm rupture were included in the study. Reasons for patients with large AAAs to remain untreated were published previously^10^.

#### Control cohort

As a control group, patients who were followed at the outpatient clinic of Vascular Surgery at Karolinska University Hospital and had a registered CTA examination between 2009 and 2013 were included. The inclusion criteria were that they had an AAA with a Dmax of between 40 and 50 mm and had been followed for at least 4 years without surgical intervention or rupture. These patients had not reached the surgical threshold for repair (55 mm for men and 52 mm for women). Details about this cohort were published previously^20^.

### Biomechanical and geometric analysis

CTA images were anonymized and extracted from the hospital archive of digital images. AAAs were segmented semi-automatically to create three-dimensional models of the AAAs, including the lumen, intraluminal thrombus, and wall. The biomechanical analysis then proceeded using finite element analysis. The segmentation process is described in detail elsewhere^21^, but is semi-automatic; the operator aids in delineating the lumen-intraluminal thrombus boundary and the outer vessel boundary, after which the geometric parameters are automatically calculated by a computer. The Dmax in this study represents an outer-to-outer wall measurement, is perpendicular to the vessel centre line and represents the Dmax in any direction, and is measured automatically from the reconstructed three-dimensional model. Aneurysm volume is defined as the entire volume of the aorta between the most inferior renal artery and the aortic bifurcation.

The biomechanical methodology modelled the AAA and the intraluminal thrombus as isotropic, hyperelastic, and incompressible^22^. The constitutive properties, vessel wall strength and wall thickness, of the model are informed by geometric characteristics and can be further adjusted according to patient characteristics (sex, age, long-term blood pressure, and family history of rupture), according to a previously published material model^23^. In this study, models were adjusted according to age and sex. It was assumed that there was no family history of AAA and the blood pressure for the simulation was set at 140/80 mmHg. Inter- and intra-observer studies have shown reliable reproducibility of the analysis^24,25^. The biomechanical software used is commercially available (A4clinics Research Edition, VASCOPS GmbH, Graz, Austria).

### Statistical analysis

In the primary statistical analysis, investigating time to rupture in relation to PWRI and PWS, aneurysms less than 70 mm were included, due to the constitutive material model used in PWRI calculations being validated for aneurysms less than 70 mm^23^. Sensitivity analyses including control patients and abdominal aortic aneurysms of all sizes were performed.

Cox proportional hazards regression was used, as implemented in the R package Survival^26^. Due to multiple observations per patient, a time-dependent Cox proportional hazards model was used, with robust standard errors to account for the interdependence of the observations from the same patient. The analysis was performed first in a univariable setting with the relevant variables (Dmax, aneurysm volume, PWS, and PWRI) and then in separate multivariable models, investigating the relationship between PWRI or PWS and time to rupture adjusted for aneurysm size (Dmax or volume) or adjusted for aneurysm size and sex. The time horizon for aneurysms included in the Cox proportional hazards regression was 10 years.

PWRI was further analysed in a Dmax-matched setting, where AAAs within 1 year, 2 years, and 4 years from rupture were classified as cases and compared with diameter-matched patients, selected from all other cases included in the study as controls. The matching was done using the R package MatchIt.

To analyse the predictive value of PWRI in addition to Dmax, two different logistic regression models that included only Dmax or Dmax together with PWRI were fitted to a binary outcome of rupture within 1 year, 2 years, or 4 years. The models were fitted to either patients who underwent a pre-rupture CTA examination and with Dmax less than 70 mm or patients irrespective of Dmax. Receiver operating characteristic (ROC) curves and the Adequacy Index^27^ were used to evaluate the added predictive value of PWRI. The Adequacy Index corresponds to the ratio of the log likelihood that is explained by the expanded model compared with the original model. An Adequacy Index of 100% corresponds to the same information in the base model as the expanded model (no added predictive value) and a lower value indicates that the expanded model provides additional predictive information. The *P* value associated with the Adequacy Index denotes a likelihood ratio test between the models.

Descriptive data are presented as median (interquartile range (i.q.r.)) or *n* (%). *P* < 0.050 was considered statistically significant. All analyses were conducted using the R programming language (version 4.21). The reporting of this study is consistent with the STROBE statement.^28^

The collection and study of patient data and radiological images was approved by the Regional Ethical Review Board in Stockholm (2016–89/31).

## Results

### Patient characteristics

**Fig. 1:**
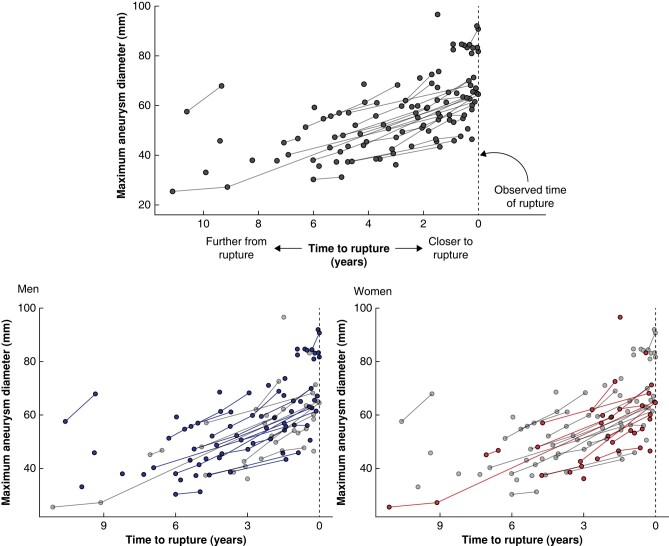
Maximum aneurysm diameter in CT angiography examinations before abdominal aortic aneurysm rupture The top panel shows all patients and the bottom panels show men and women separately. Each circle represents a CT angiography examination and lines join up circles for the same patient.

Sixty-seven patients who underwent 109 CTA examinations before aneurysm rupture were identified and included; 47 (70%) were male and the median age at the time of CTA examination was 77 (i.q.r. 71–83) years. The median Dmax was 56 (i.q.r. 46–65) mm and the median time to rupture was 2.13 (i.q.r. 0.64–4.72) years (*Table 1* and *Fig. 1*). Of the CTA examinations that were within a year from rupture, six (18%) AAAs were less than 55 mm, and, among those that were within 1 to 2 years from rupture, seven (35%) AAAs were less than 55 mm (*Table S1*). *Figure S2* shows PWRI and PWS in relation to time to rupture.

**Table 1:** Patient and aneurysm characteristics for the pre-rupture cohort and the stable cohort

	Pre-rupture cohort, *n* (of patients) = 67	Stable cohort, *n* (of patients) = 97	P*
Age (years), median (i.q.r.)	77 (71–83)	72 (66–77)	0.004
**Sex**			0.0270
Male	47 (70)	82 (85)	
Female	20 (30)	15 (15)	
**Diabetes**	10 (16)	15 (15)	0.980
Unknown, *n*	3	0	
**Smoking**			0.032
Current	13 (34)	25 (26)	
Previous	13 (34)	55 (58)	
Never	12 (32)	15 (16)	
Unknown, *n*	29	2	
**Height (m), median (i.q.r.)**	1.73 (1.62–1.76)	1.75 (1.69–1.80)	0.053
Unknown, *n*	34	4	
**Weight (kg), median (i.q.r.)**	76 (63–80)	80 (67–88)	0.120
Unknown, *n*	33	5	
**Dmax at rupture (mm), median (i.q.r.)**	74 (64–83)	–	
Unknown, *n*	28	–	

	Pre-rupture cohort, *n* (of CTA examinations) = 109	Stable cohort, *n* (of CTA examinations) = 97	P*
Time to rupture/censoring (years), median (i.q.r.)	2.13 (0.64–4.72)	5.26 (4.11–6.59)	<0.001
Dmax (mm), median (i.q.r.)	56 (46–65)	46 (43–47)	<0.001
Aneurysm volume (cm^3^), median (i.q.r.)	168 (104–236)	95 (83–115)	<0.001
Peak wall stress (kPa), median (i.q.r.)	193 (155–238)	174 (153–187)	<0.001
Peak wall rupture index (ratio), median (i.q.r.)	0.39 (0.28–0.50)	0.31 (0.27–0.36)	<0.001

Values are *n* (%) unless otherwise indicated. The cohorts are presented at patient level and then at CTA level. i.q.r., interquartile range; Dmax, maximum aneurysm diameter. *Wilcoxon rank sum test; Pearson’s chi-squared test.

In the control cohort, 97 patients were included. These patients were younger than the patients in the pre-rupture cohort (median age of 72 (i.q.r. 66–77) years; *P* = 0.004) and were more often male (82, 85%; *P* = 0.027). When comparing measurements from CTA examinations between the pre-rupture group and the control group, Dmax, aneurysm volume, PWS, and PWRI were all lower in the control group (*P* < 0.001 for all) (*Table 1*).

### Factors associated with risk of rupture

A survival model was used to investigate the association between biomechanical parameters and risk of rupture. The association was further adjusted for potential confounders: aneurysm size (Dmax or volume) and sex. In the primary survival analysis, only pre-rupture AAAs were included and large aneurysms (greater than 70 mm) were excluded; in total, there were 53 patients who underwent 91 CTA examinations who had a Dmax less than or equal to 70 mm.

In the univariable analysis, Dmax had an HR of 1.05 (95% c.i. 1.02 to 1.09) per mm for rupture. Aneurysm volume, PWS, and PWRI also had significant associations with risk of rupture (*Table 2*). The results were similar when adjusting for sex (*Table S2*). In the analyses of Dmax and aneurysm volume, women had an increased risk of rupture (HR 2.16, 95% c.i. 1.23 to 3.78 (*P* = 0.007) and HR 1.92, 95% c.i. 1.06–3.50 (*P* = 0.033) respectively).

**Table 2:** Cox proportional hazards analysis: univariable association between time to rupture and geometric and biomechanical indices of intact abdominal aortic aneurysms less than 70 mm, with a known time to rupture

	HR (95% c.i.)	*P*	HR (95% c.i.)	*P*	HR (95% c.i.)	*P*	HR (95% c.i.)	*P*
Dmax (mm)	1.05 (1.02, 1.09)	<0.001*	–	–	–	–	–	–
Aneurysm volume (cm^3^)	–	–	1.01 (1.00, 1.01)	<0.001*	–	–	–	–
Peak wall stress (kPa)	–	–	–	–	1.01 (1.01, 1.01)	<0.001*	–	–
Peak wall rupture index (%)	–	–	–	–	–	–	1.05 (1.04, 1.07)	<0.001*
R^2^	0.206		0.211		0.153		0.236	
BIC	304		303		310		300	
AIC	302		301		308		298	
C-index	0.733		0.740		0.686		0.702	

^*^Statistically significant. Dmax, maximum aneurysm diameter; BIC, Bayesian information criterion; AIC, Akaike information criterion.

In the multivariable analysis including a variable for aneurysm size, PWRI was associated with risk of rupture (*Table 3*). In the model including Dmax, the HR for PWRI was 1.05 (95% c.i. 1.03 to 1.08) per % (*P* < 0.001), and, when including aneurysm volume, the HR for PWRI was 1.05 (95% c.i. 1.02 to 1.08) per % (*P* < 0.001). Adjusting these multivariable models for sex did not change the results (*Table S3*). PWS was not significantly associated with risk of rupture in the analysis including size or in the analysis including size and sex. When adjusting models for smoking history (ever smoker), smoking conferred an increased hazard for rupture in the models that were adjusted for Dmax and PWRI or PWS (HR 2.92 (*P* = 0.019) and HR 3.92 (*P* = 0.015) respectively) (*Table S4*) and PWRI remained significantly associated with rupture.

**Table 3:** Cox proportional hazards analysis: multivariable association between rupture and biomechanical indices of intact abdominal aortic aneurysms less than 70 mm, with a known time to rupture, adjusted for aneurysm size (Dmax or volume)

	HR (95% c.i.)	*P*	HR (95% c.i.)	*P*	HR (95% c.i.)	*P*	HR (95% c.i.)	*P*
Peak wall rupture index (%)	1.05 (1.03, 1.08)	<0.001*	1.05 (1.02, 1.08)	<0.001*	–	–	–	–
Peak wall stress (kPa)	–	–	–	–	1.00 (1.00, 1.01)	0.340	1.00 (1.00, 1.01)	0.390
Dmax (mm)	1.10 (1.06, 1.14)	<0.001*	–	–	1.12 (1.09, 1.16)	<0.001*	–	–
Aneurysm volume (cm^3^)	–	–	1.01 (1.01, 1.01)	<0.001*	–	–	1.01 (1.01, 1.02)	<0.001*
R^2^	0.345		0.338		0.301		0.298	
BIC	404		406		417		417	
AIC	400		402		413		413	
C-index	0.778		0.792		0.773		0.789	

^*^Statistically significant. Dmax, maximum aneurysm diameter; BIC, Bayesian information criterion; AIC, Akaike information criterion.

### Sensitivity analyses including non-ruptured AAAs and AAAs of all sizes

As a sensitivity analysis, a control cohort of 97 patients with non-ruptured AAAs that remained stable were included. The inclusion of these stable patients in the survival analysis did not qualitatively change the results, but point estimates were different (*Table S5*).

A sensitivity analysis that included AAAs of all sizes was also undertaken (*Table S6*); in this analysis, there was a trend for a significant HR for time to rupture for PWS when including aneurysm volume (HR 1.00, 95% c.i. 1.00 to 1.01; *P* = 0.060), but not aneurysm diameter. There was no association between PWRI and time to rupture. However, both models including PWRI violated the proportional hazards assumption.

Further, in a case–control setting, pre-rupture patients were classified as having an AAA rupture within 4 years, 2 years, or 1 year and were considered as cases when rupture occurred in each interval; patients who did not experience AAA rupture within each interval, as well as all control patients, were used as controls in a Dmax-matching procedure. This yielded three sets of Dmax-matched cohorts of 29 *versus* 29 patients, 20 *versus* 20 patients, and 8 *versus* 8 patients respectively. Multivariable logistic regression was used to account for residual confounding of Dmax. For the 4-year outcome, PWRI was associated with pre-rupture status (OR 1.13; *P* = 0.008). For the 2-year outcome, there was a trend (OR 1.08; *P* = 0.079), and, for the 1-year outcome, no association was seen (OR 1.03; *P* = 0.686) (*Fig. S3* and *Table S7*).

### Added value of peak wall rupture index to semi-automatic diameter for prediction of rupture within 1 year, 2 years, and 4 years

To examine the added predictive value of PWRI in addition to Dmax to predict rupture within 1 year, 2 years, and 4 years, logistic regression models were fitted with either Dmax or Dmax and PWRI, to predict a binary outcome of rupture within 1 year, 2 years, or 4 years. Models were fitted to two data sets, either all patients who underwent a pre-rupture CTA examination or patients who underwent a pre-rupture CTA examination and with an AAA less than 70 mm.

**Fig. 2:**
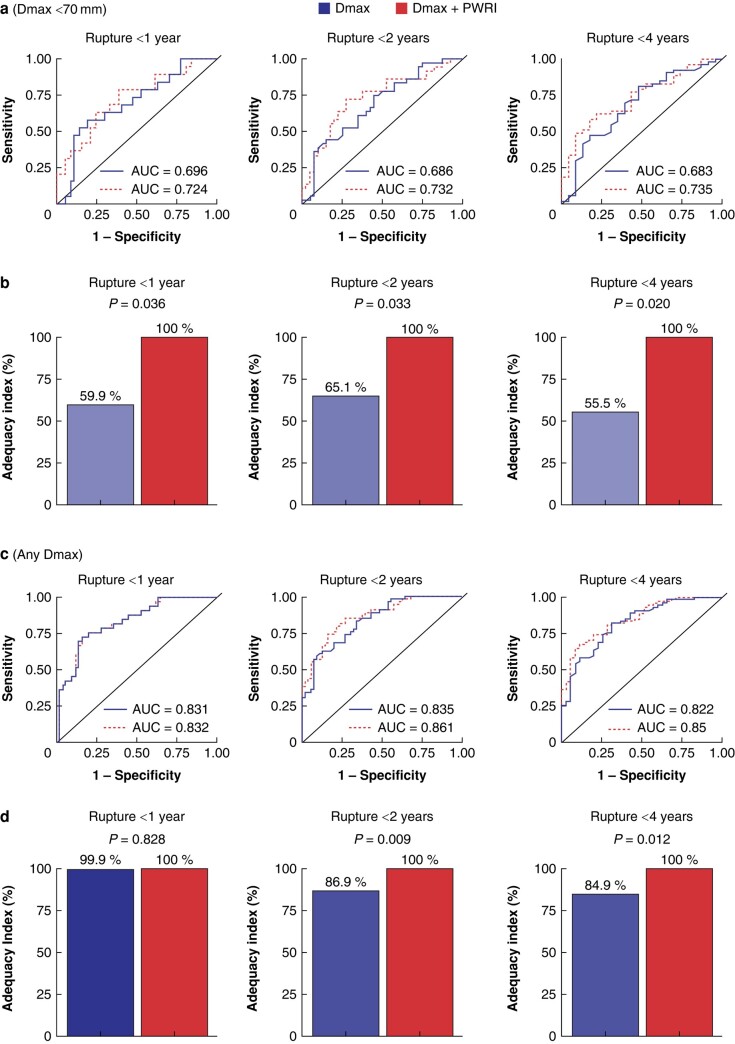
Patients with abdominal aortic aneurysms less than 70 mm (a, b) or of any Dmax (c, d), with a known time to rupture, classified in a binary fashion, according to time to rupture (within 1 year, within 2 years, or within 4 years) Two different logistic regression models are compared for the outcome of belonging to the pre-rupture group within the specified time frame. The base model includes only Dmax and the expanded model includes Dmax and PWRI. Two different performance measures are compared: receiver operating characteristic (ROC) curves for the different time points (**a**, **c**); and the Adequacy Index (**b**, **d**). Dmax, maximum aneurysm diameter; PWRI, peak wall rupture index.

In patients with an AAA and Dmax less than 70 mm (*Fig. 2a*,*b*), the area under the curve (AUC) was numerically higher with the model including both Dmax and PWRI, for rupture within 1 year (0.724 *versus* 0.696), rupture within 2 years (0.732 *versus* 0.686), and rupture within 4 years (0.735 *versus* 0.683). Models that only included Dmax had an Adequacy Index of 55.5–65.5% of the model that also included PWRI, a model with a reference Adequacy Index of 100%. The likelihood ratio test was significant for all models. In patients with AAAs of any Dmax (*Fig. 2c*,*d*), the differences in AUC were smaller and the Adequacy Index indicated improvement that was significant for the outcomes of rupture within 2 and 4 years (*Tables S8*, *S9*). The highest specificity that could be achieved (with 100% sensitivity) for the model with Dmax alone (for rupture within 1 year, 2 years, and 4 years) was 0.288, 0.125, and 0.0435 respectively compared with 0.158, 0.0750, and 0.130 respectively for the model including PWRI, for AAAs less than 70 mm. For AAAs of all sizes, the corresponding numbers were 0.368, 0.357, and 0.171 respectively for the models that only included Dmax compared with 0.355, 0.321, and 0.286 respectively for the model that also included PWRI.

## Discussion

This study, based on biomechanical factors derived from clinical imaging for patients with at least one CTA examination before rupture, showed an association between PWRI and risk of rupture of small and medium AAAs when adjusted for aneurysm size and/or sex. Women suffer an increased rupture risk given the same size of AAA.

Studies in the field of biomechanical analysis have largely focused on differences between ruptured and non-ruptured AAAs, as summarized in three recent meta-analyses^16–18^. When comparing diameter-matched AAAs, PWRI, but not PWS, was increased in ruptured AAAs. The analysis of ruptured AAAs compared with stable AAAs does not correspond to the true clinical situation and the rupture itself may affect aneurysm shape and thereby influence the estimated biomechanical parameters. Two retrospective studies have specifically investigated biomechanical rupture prediction for AAAs before rupture^19,20^. They included 13 and 19 patients before rupture respectively and PWRI was indicated as a predictor, independent of Dmax. These studies have analysed patients with times categorized at group level, but did not utilize survival analysis methods. In the prospective setting, PWS and PWRI have been associated with future aneurysm events^29^, rupture or symptoms^30^, and progression to surgery^31^.

In this study, PWRI, but not PWS, was associated with time to rupture. A single wall stress value, such as PWS, may not be enough to predict AAA rupture, as an AAA may have several regions of increased wall stress. PWRI, on the other hand, is a more comprehensive biomechanical feature of an aneurysm, which may provide more applicable rupture risk information. In the current implementations of PWRI, the wall strength model is extrapolated from patient characteristics and geometric properties. Future work in imaging and/or aneurysm biology may provide quantitative biomarkers that can inform a more specific wall strength model, for instance the combination of CT and MRI has been suggested to measure patient-specific wall thickness^32^.

In the present analysis, it appears that PWRI adds predictive value specifically in the case of medium or small AAAs (less than 70 mm). This is concordant with previous results from Singh *et al*.^33^, where, for large diameter-matched AAAs, they found no difference in PWRI, but a trend towards increased PWS, among patients with ruptured AAAs, which is similar to the findings of the diameter-unrestricted sensitivity analysis of the present study.

It is known that women suffer from rupture at smaller diameters than men and this is something that the results of the present study support, with HRs of approximately 2 for women when adjusting for aneurysm size. When adjusting for sex, the association between PWRI and rupture risk is weaker, but still statistically significant, compared with when not adjusting for sex. This indicates that part of the effect of PWRI on rupture risk may be explained by sex and PWRI factors related to sex, namely decreased wall strength and increased wall stress^23,34^.

It should be noted that the size adjustment in this study is by semi-automatically measured aneurysm diameter or aneurysm volume. The authors have previously shown that this method of measuring the Dmax may be more sensitive at detecting aneurysm-related outcomes compared with traditionally used clinical diameter measurements^30^. Therefore, this method of measuring size probably provides a more powerful adjustment when compared with routine clinical Dmax measurements.

There are multiple reasons why a patient with a known aneurysm experiences aneurysm rupture. It has been previously presented from part of this cohort that a third of ruptures occurred in known aneurysms^10^. The main reasons for this were: that patients were denied surgery; that AAAs were missed in surveillance; patient choice; or size related. However, survival after intervention for the AAA rupture was similar in all groups. This points to an area of potential improvement in the management of patients with AAAs. The authors’ results show that, in the aneurysms that do rupture, a non-negligible proportion are below 55 mm (9% within 6 months from rupture and 36% within 6 months to 1 year from rupture). It should further be noted that these represent diameters measured using CTA, which are known to be larger compared with diameters measured using ultrasonography^35^.

It is difficult to find a properly matched control group with non-eventful follow-up, as most patients are treated in accordance with guidelines. This can truly only be addressed with a prospective cohort study design. In the main analysis, the rupture time of all AAAs is known and no patients are censored to due surgery or death. However, it is not certain whether all AAAs would eventually rupture given unlimited follow-up or whether the aneurysms that rupture represent a rupture-prone phenotype.

In the biomechanical model, patient-specific blood pressure and heredity were not included in the biomechanical analysis. Unfortunately, it is difficult to collect and verify accurate blood pressure readings in a retrospective cohort, especially those that would be representative of the time for the imaging examinations. Further, family history was also not included in the model, as it is difficult to evaluate the reliability of these data. These two factors influence the biomechanical computation, but are likely to improve rupture risk prediction when included. Further, the results are still generalizable, as these two parameters can be left out from the analysis even when they are known. Some patients with ruptured AAAs die before reaching the hospital and are never diagnosed. These patients could not be included in this study. In the primary analyses of biomechanical variables, the aneurysms are limited to a size of 70 mm, limiting the applicability to larger aneurysms, but the postponement of repair of such large aneurysms is not primarily due to factors related to the aneurysm and rupture risk, but due to the patient. The statistical treatment does not consider the growth of the morphological variables, or increase in the biomechanical variables, but instead treats them as constant (until the next observation). A larger cohort with more frequent surveillance could allow for more a comprehensive multivariate model, where the predictors are modelled simultaneously and used to inform the survival model (a so-called joint model)^36^.

This report on CT findings detected in patients prior to their later aneurysm rupture supports that an increased PWRI is associated with increased rupture risk. The results also confirm the previously reported higher rupture risk in women, given the same size of AAA, and it reinforces the need for a sex-specific intervention threshold. Our results support the notion of considering the entire geometry of an aneurysm, rather than sole reliance on maximum diameter measurements.

## Supplementary Material

znae125_Supplementary_Data

## Data Availability

Individual-level data and images from patient healthcare records are not publicly available as they constitute sensitive information.
