# Supplementary material for: Peak wall rupture index is associated with risk of rupture of abdominal aortic aneurysms, independent of size and sex
Source: Br J Surg. 2024 May 24;111(5):znae125. doi: 10.1093/bjs/znae125 (PMC11116082; doi:10.1093/bjs/znae125)
Supplement: znae125_Supplementary_Data [file znae125_supplementary_data.docx]

**Supplementary material**

**
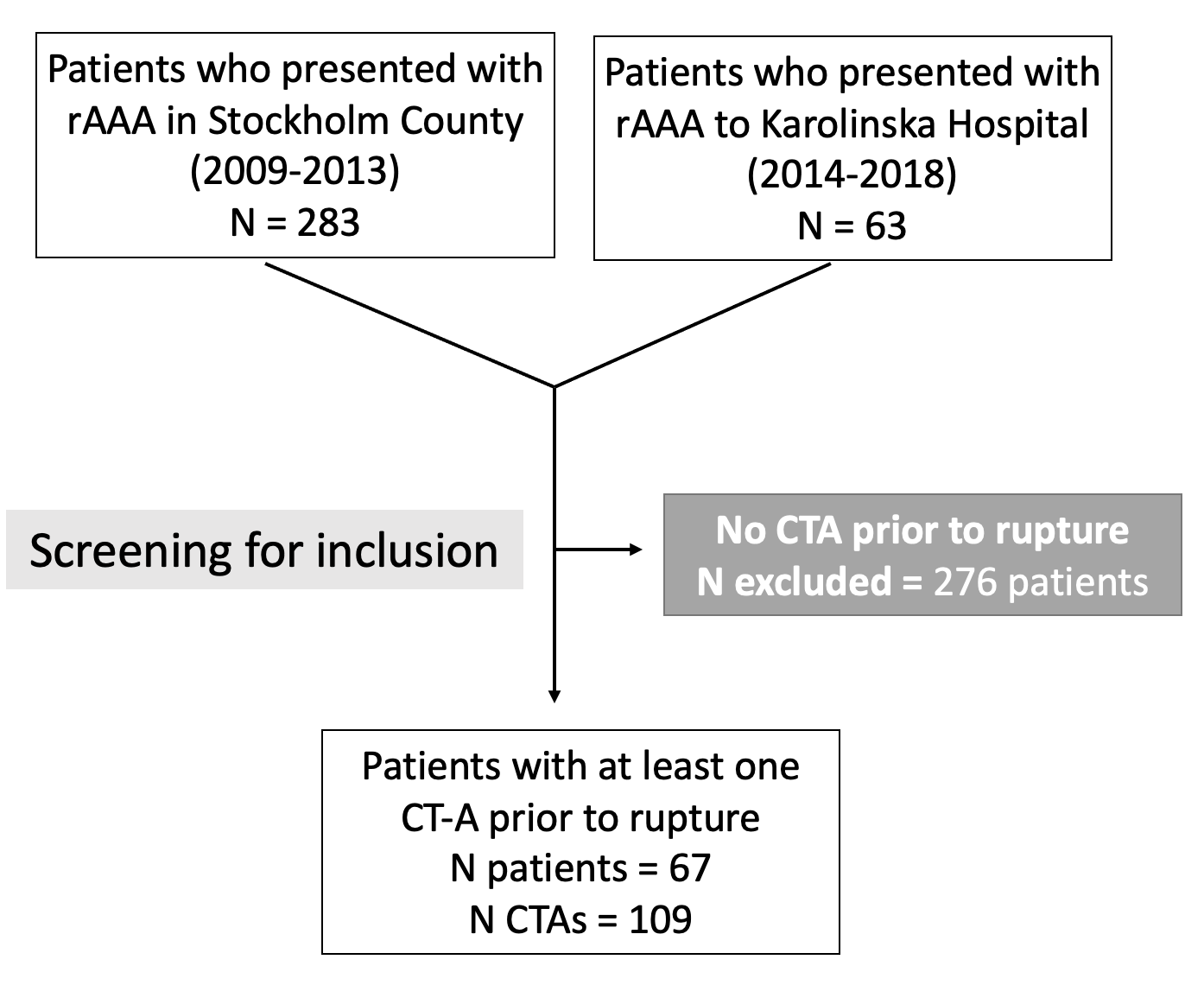
**

**Supplementary Figure 1.** Flow chart of inclusion of patients into the study. rAAA = ruptured abdominal aortic aneurysm; CTA= computed tomography angiography


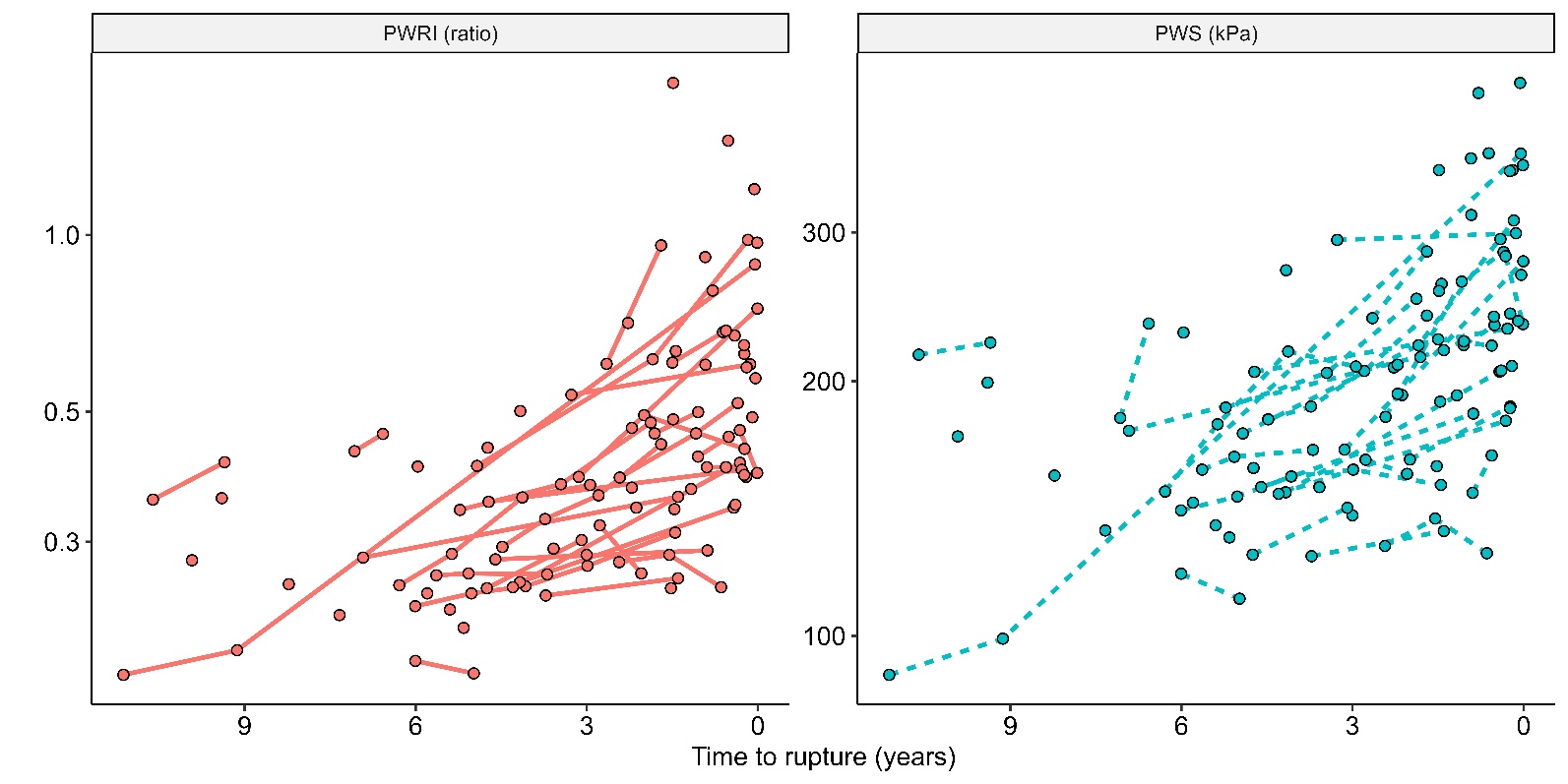


Supplementary Figure 2. Biomechanical indices in relation to time-to-rupture among patients that have a known time until rupture. (Left) PWRI = Peak Wall Rupture Index, ratio, (Right) PWS = Peak Wall Stress, kPa.

**Supplementary Table 1.** Maximal aneurysm diameter of CTAs prior to rupture, grouped according to time-to-rupture (years); maximal aneurysm diameter, median (IQR), min and max, and number below 55 mm. Data presented on CTA level.

| **Time**  **Interval (years)** | **N of CTs** | **Dmax (mm)** ^1^ | **Min**  **Dmax (mm)** | **Max**  **Dmax (mm)** | **Smaller**  **than 55**  **mm (n)** | **Aneurysm**  **volume (cm3)** ^1^ | **PWRI**  **(ratio)** ^1^ | **PWS**  **(kPa)** ^1^ |
| --- | --- | --- | --- | --- | --- | --- | --- | --- |
| <1 | 33 | 65.6  (60.1-83.2) | 45.9 | 92.0 | 6 (18.2) | 281.8  (185.8-417.8) | 0.57  (0.4-0.69) | 240.5  (205.9-314.6) |
| 1-2 | 20 | 58  (53.7-65.8) | 43.4 | 96.7 | 7 (35) | 175.3  (133.5-229.4) | 0.46  (0.35-0.53) | 220.6  (182.3-251.8) |
| 2-4 | 21 | 52.3  (43.6-59.9) | 36.2 | 71.1 | 11 (52.4) | 130  (106.1-193.9) | 0.34  (0.28-0.39) | 181.5  (155.4-205.7) |
| 4-6 | 21 | 47.3  (41.4-55.8) | 31.2 | 68.6 | 15 (71.4) | 92.6  (80.3-167.9) | 0.26  (0.25-0.35) | 157.2  (146.1-180.3) |
| 6-8 | 7 | 40.3  (38-45.9) | 30.3 | 51.3 | 7 (100) | 85.9  (66.3-133.2) | 0.25  (0.23-0.36) | 148.1  (136.9-177.9) |
| >8 | 7 | 38  (30.1-51.7) | 25.5 | 67.9 | 5 (71.4) | 82.9  (49.8-142.6) | 0.28  (0.22-0.36) | 172.1  (126.9-207.2) |
| ^1^Presented as median (IQR). Abbreviations: PWRI = peak wall rupture index, PWS = peak wall stress, Dmax = Maximal Aneurysm Diameter. | | | | | | | | |

**Supplementary Table 2.** Time-to-rupture analysis including pre-rupture patients with geometric and biomechanical parameters and sex. Models include sex and a size- or biomechanical variable.

| **Characteristic** | **HR** **(95% CI)**^1^ | **p-value** | **HR** **(95% CI)**^1^ | **p-value** | **HR** **(95% CI)**^1^ | **p-value** | **HR** **(95% CI)**^1^ | **p-value** |
| --- | --- | --- | --- | --- | --- | --- | --- | --- |
| Sex |  |  |  |  |  |  |  |  |
| Male | - | - | - | - | - | - | - | - |
| Female | 2.16  (1.23 to 3.78) | **0.007** | 1.92  (1.06 to 3.50) | **0.033** | 1.74  (0.87 to 3.50) | 0.12 | 1.02  (0.51 to 2.03) | 0.97 |
| Dmax (mm) | 1.06  (1.03 to 1.09) | **<0.001** | - | - | - | - | - | - |
| Aneurysm vol (cm3) | - | - | 1.01  (1.00 to 1.01) | **<0.001** | - | - | - | - |
| PWS (kPa) | - | - | - | - | 1.01  (1.01 to 1.01) | **<0.001** | - | - |
| PWRI (ratio) | - | - | - | - | - | - | 1.05  (1.04 to 1.07) | **<0.001** |
| R² | 0.254 |  | 0.248 |  | 0.182 |  | 0.236 |  |
| BIC | 302 |  | 303 |  | 311 |  | 304 |  |
| AIC | 298 |  | 299 |  | 307 |  | 300 |  |
| c-index | 0.740 |  | 0.759 |  | 0.696 |  | 0.704 |  |
| ^1^HR = Hazard Ratio, CI = Confidence Interval. BIC = Bayesian information criterion, AIC = akaike information criterion | | | | | | | | |

**Supplementary Table 3.** Multivariable association between time-to-rupture and geometric and biomechanical indices of intact abdominal aortic aneurysms less than 70 mm, with a known time-to-rupture. Models include a biomechanical variable, a variable for size and sex.

|  | **HR**  **(95% CI)**^1^ | **p-value** | **HR**  **(95% CI)**^1^ | **p-value** | **HR**  **(95% CI)**^1^ | **p-value** | **HR**  **(95% CI)**^1^ | **p-value** |
| --- | --- | --- | --- | --- | --- | --- | --- | --- |
| PWRI (ratio) | 1.03  (1.00 to 1.06) | **0.021** | 1.03  (1.01 to 1.05) | **0.011** | - | - | - | - |
| PWS (kPa) | - | - | - | - | 1.00  (1.00 to 1.01) | 0.39 | 1.00  (1.00 to 1.01) | 0.19 |
| Dmax (mm) | 1.04  (1.00 to 1.08) | **0.049** | - | - | 1.05  (1.01 to 1.10) | **0.020** | - | - |
| Aneurysm vol (cm3) | - | - | 1.01  (1.00 to 1.01) | **0.007** | - | - | 1.01  (1.00 to 1.01) | **0.001** |
| Sex |  |  |  |  |  |  |  |  |
| Female | 1.48  (0.78 to 2.79) | 0.23 | 1.37  (0.70 to 2.67) | 0.36 | 2.08  (1.17 to 3.69) | **0.012** | 1.89  (1.01 to 3.54) | **0.046** |
| Male | — |  | — |  | — |  | — |  |
| R² | 0.280 |  | 0.280 |  | 0.259 |  | 0.257 |  |
| BIC | 303 |  | 303 |  | 306 |  | 306 |  |
| AIC | 297 |  | 297 |  | 300 |  | 300 |  |
| c-index | 0.742 |  | 0.751 |  | 0.744 |  | 0.752 |  |
| ^1^HR = Hazard Ratio, CI = Confidence Interval. Abbreviations: PWRI = peak wall rupture index, PWS = peak wall stress, Dmax = Maximal Aneurysm Diameter. BIC = Bayesian information criterion, AIC = akaike information criterion | | | | | | | | |

**Supplementary Table 4.** Multivariable association between time-to-rupture and geometric and biomechanical indices of intact abdominal aortic aneurysms less than 70 mm, with a known time-to-rupture. Models include a biomechanical variable, a variable for size and smoking status (ever smoker).

|  | **HR** **(95% CI)**^1^ | **p-value** | **HR** **(95% CI)**^1^ | **p-value** | **HR** **(95% CI)**^1^ | **p-value** | **HR** **(95% CI)**^1^ | **p-value** |
| --- | --- | --- | --- | --- | --- | --- | --- | --- |
| PWRI (ratio) | 1.05  (1.01 to 1.08) | **0.005** | 1.04  (1.01 to 1.08) | **0.014** | - | - | - | - |
| PWS (kPa) | - | - | - | - | 1.01  (1.00 to 1.02) | **0.002** | 1.01  (1.00 to 1.02) | 0.075 |
| Dmax (mm) | 1.05  (1.00 to 1.10) | 0.062 | - | - | 1.05  (0.99 to 1.10) | 0.092 | - | - |
| Aneurysm vol (cm3) | - | - | 1.00  (1.00 to 1.01) | 0.10 |  |  | 1.00  (1.00 to 1.01) | 0.20 |
| Smoking |  |  |  |  |  |  |  |  |
| Never smoker | - | - | - | - | - | - | - | - |
| Ever Smoker | 2.92  (1.19 to 7.15) | **0.019** | 2.10  (0.90 to 4.89) | 0.087 | 3.92  (1.30 to 11.8) | **0.015** | 2.57  (0.82 to 8.04) | 0.11 |
| ^1^HR = Hazard Ratio, CI = Confidence Interval | | | | | | | | |

**Supplementary Table 5.** Time-to-rupture analysis including pre-rupture patients and patients that are stable (censored at the end of follow-up). Biomechanical variables (PWRI and PWS) adjusted for size (Dmax or aneurysm volume) and sex.

| **Characteristic** | **HR**  **(95% CI)**^1^ | **p-value** | **HR**  **(95% CI)**^1^ | **p-value** | **HR**  **(95% CI)**^1^ | **p-value** | **HR**  **(95% CI)**^1^ | **p-value** |
| --- | --- | --- | --- | --- | --- | --- | --- | --- |
| PWRI (ratio) | 1.03  (1.00 to 1.06) | **0.043** | 1.03  (1.00 to 1.06) | **0.034** | - | - | - | - |
| PWS (kPa) | - | - | - | - | 1.00  (1.00 to 1.01) | 0.43 | 1.00  (1.00 to 1.01) | 0.29 |
| Dmax (mm) | 1.12  (1.07 to 1.17) | **<0.001** | - | - | 1.13  (1.08 to 1.18) | **<0.001** | - | - |
| Aneurysm Vol  (cm3) | - | - | 1.01  (1.01 to 1.02) | **<0.001** | - | - | 1.01  (1.01 to 1.02) | **<0.001** |
| Sex | - | - | - | - | - | - | - | - |
| Male | - | - | - | - | - | - | - | - |
| Female | 2.72  (1.37 to 5.38) | **0.004** | 2.56  (1.32 to 4.96) | **0.005** | 3.86 (2.19 to 6.79) | **<0.001** | 3.54  (1.96 to 6.39) | **<0.001** |
| ^1^HR = Hazard Ratio, CI = Confidence Interval. Abbreviations: PWRI = peak wall rupture index, PWS = peak wall stress, Dmax = Maximal Aneurysm Diameter. | | | | | | | | |

**Supplementary Table 6.** AAAs with no diameter restriction, only including patients with CTAs prior to rupture. Cox PH models of time-to-rupture, including PWS or PWRI and a parameter for size (maximal aneurysm diameter or volume)

| **Characteristic** | **HR**  **(95% CI)**^1^ | **p-value** | **HR**  **(95% CI)**^1^ | **p-value** | **HR**  **(95% CI)**^1^ | **p-value** | **HR**  **(95% CI)**^1^ | **p-value** |
| --- | --- | --- | --- | --- | --- | --- | --- | --- |
| PWRI (ratio) | 1.00  (0.99 to 1.01) | 0.95 | 1.00  (0.99 to 1.01) | 0.75 | - | - | - | - |
| PWS (kPa) | - | - | - | - | 1.00  (1.00 to 1.01) | 0.11 | 1.00  (1.00 to 1.01) | 0.060 |
| Dmax (mm) | 1.06  (1.03 to 1.09) | <0.001 | - | - | 1.05  (1.01 to 1.08) | 0.005 | - | - |
| Aneurysm vo  l (cm3) | - | - | 1.01  (1.00 to 1.01) | <0.001 | - | - | 1.01  (1.00 to 1.01) | <0.001 |
| ^1^HR = Hazard Ratio, CI = Confidence Interval. Abbreviations: PWRI = peak wall rupture index, PWS = peak wall stress, Dmax = Maximal Aneurysm Diameter. | | | | | | | | |


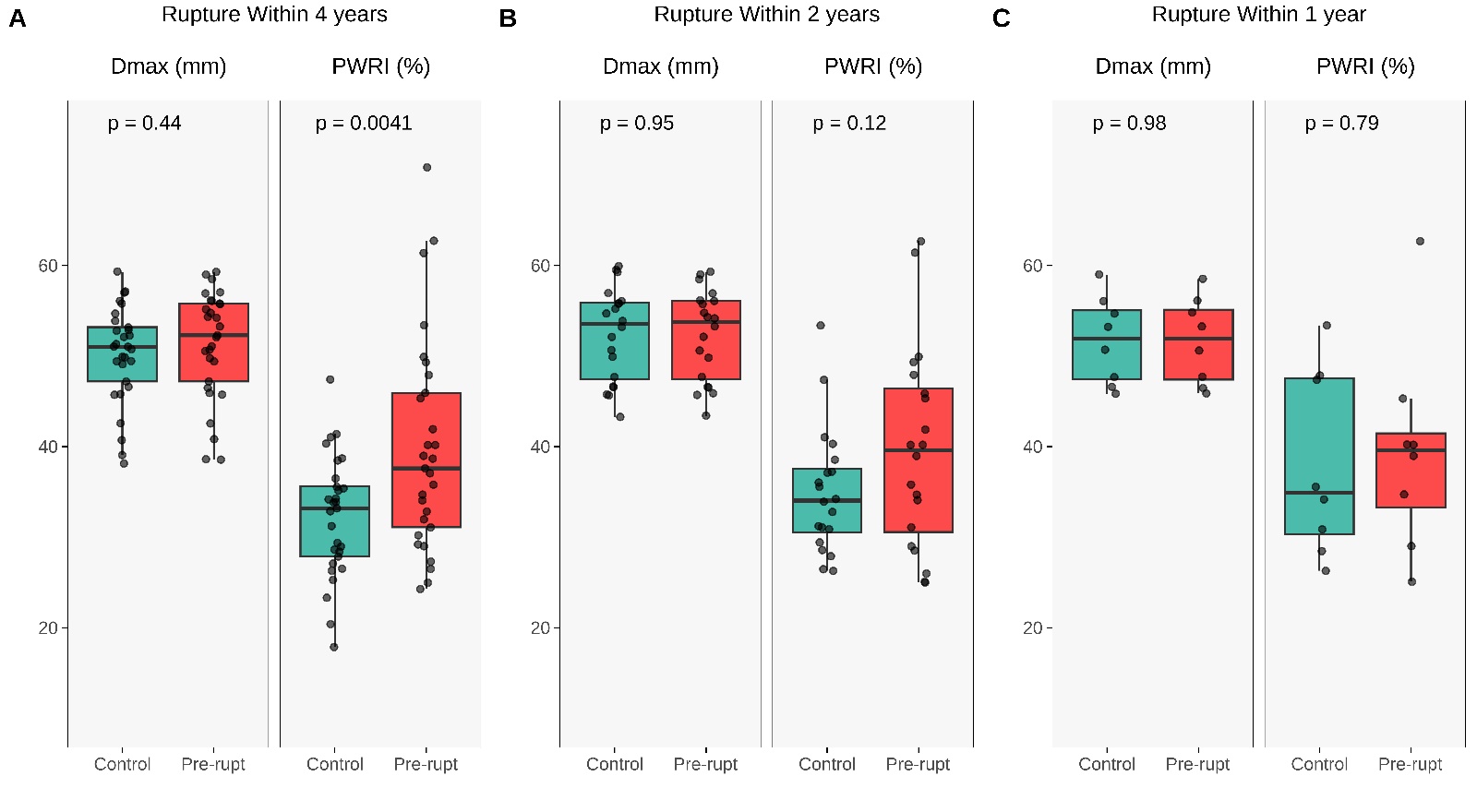


**Supplementary Figure 3.** Matched analysis of pre-rupture patients that ruptured within the specified time frame (A) 4 years, (B) 2 years (C) 1 year, matched against controls (not rupture within that time frame, or part of the control cohort) by maximal aneurysm diameter. P-values denotes a t-test.

**Supplementary Table 7.** Table showing logistic regressions models testing association of PWRI to status of belonging to the pre-rupture group within the specified timeframe .

|  | **Rupture within**  **4 yrs**  N = 29 vs 29 | | **Rupture within**  **2 yrs**  N = 20 vs 20 | | **Rupture within**  **1 yr**  N = 8 vs 8 | |
| --- | --- | --- | --- | --- | --- | --- |
| *Predictors* | Odds Ratios | p | Odds Ratios | p | Odds Ratios | p |
| Maximal Aneurysm  Diameter (mm) | 0.94 | 0.295 | 0.93 | 0.345 | 0.96 | 0.783 |
| Peak Wall Rupture  Index (%, PWRI) | 1.13 | **0.008** | 1.08 | 0.079 | 1.03 | 0.689 |
| Observations | 58 | | 40 | | 16 | |
| ^1^OR = Odds Ratio, CI = Confidence Interval. Abbreviations: PWRI = peak wall rupture index, Dmax = Maximal Aneurysm Diameter. | | | | | | |

**Supplementary Table 8.** Table showing logistic regressions models from Figure 2, models for outcome of rupture within one, two or four years, including either Dmax or Dmax and PWRI as independent variables. Models are fitted on all pre-rupture patients with Dmax < 70 mm.

|  | | **Dmax** | | **Dmax + PWRI** | |
| --- | --- | --- | --- | --- | --- |
| **Outcome** |  | **OR** **(95% CI)**^1^ | **p-value** | **OR** **(95% CI)**^1^ | **p-value** |
| **Rupture**  **within 1 yr** | Dmax (mm) | 1.10  (1.02 to 1.19) | **0.016** | 1.05  (0.97 to 1.15) | 0.25 |
|  | PWRI (%) | - | - | 1.05  (1.00 to 1.10) | **0.045** |
| **Rupture**  **within 2 yrs** | Dmax (mm) | 1.09  (1.03 to 1.17) | **0.006** | 1.05  (0.98 to 1.13) | 0.17 |
|  | PWRI (%) | - | - | 1.05  (1.00 to 1.11) | **0.048** |
| **Rupture**  **within 4 yrs** | Dmax (mm) | 1.09  (1.02 to 1.17) | **0.013** | 1.04  (0.96 to 1.13) | 0.36 |
|  | PWRI (%) | - | - | 1.07  (1.01 to 1.15) | **0.039** |
| ^1^OR = Odds Ratio, CI = Confidence Interval. Abbreviations: PWRI = peak wall rupture index, Dmax = Maximal Aneurysm Diameter. | | | | | |

**Supplementary Table 9.** Table showing logistic regressions models from Figure 2, models for outcome of rupture within one, two or four years, including either Dmax or Dmax and PWRI as independent variables. Models are fitted on all pre-rupture patients.

|  | | **Dmax** | | **Dmax + PWRI** | |
| --- | --- | --- | --- | --- | --- |
| **Outcome** |  | **OR** **(95% CI)**^1^ | **p-value** | **OR** **(95% CI)**^1^ | **p-value** |
| **Rupture**  **within 1 yr** | Dmax (mm) | 1.11  (1.07 to 1.16) | **<0.001** | 1.10  (1.05 to 1.17) | **<0.001** |
|  | PWRI (%) | - | - | 1.00  (0.98 to 1.04) | 0.83 |
| **Rupture**  **within 2 yrs** | Dmax (mm) | 1.13  (1.08 to 1.19) | **<0.001** | 1.08  (1.02 to 1.15) | **0.011** |
|  | PWRI (%) | - | - | 1.06  (1.01 to 1.12) | **0.020** |
| **Rupture**  **within 4 yrs** | Dmax (mm) | 1.12  (1.07 to 1.18) | **<0.001** | 1.06  (1.00 to 1.13) | 0.055 |
|  | PWRI (%) | - | - | 1.08  (1.01 to 1.16) | **0.028** |
| ^1^OR = Odds Ratio, CI = Confidence Interval. Abbreviations: PWRI = peak wall rupture index, Dmax = Maximal Aneurysm Diameter. | | | | | |
